# Molecular Surface of JZTX-V (β-Theraphotoxin-Cj2a) Interacting with Voltage-Gated Sodium Channel Subtype Na_V_1.4

**DOI:** 10.3390/toxins6072177

**Published:** 2014-07-23

**Authors:** Ji Luo, Yiya Zhang, Mengting Gong, Shanshan Lu, Yifeng Ma, Xiongzhi Zeng, Songping Liang

**Affiliations:** The key Laboratory of Protein Chemistry and Developmental Biology of Ministry of Education, College of Life Science, Hunan Normal University, Changsha 410081, China; E-Mails: luoji927@gmail.com (J.L.); yiya-180@163.com (Y.Z.); gmt20130219@hotmail.com (M.G.); susan880102@163.com (S.L.); 15111431824@163.com (Y.M.)

**Keywords:** spider toxin, voltage gated sodium channels, JZTX-V, Na_V_1.4

## Abstract

Voltage-gated sodium channels (VGSCs; Na_V_1.1–Na_V_1.9) have been proven to be critical in controlling the function of excitable cells, and human genetic evidence shows that aberrant function of these channels causes channelopathies, including epilepsy, arrhythmia, paralytic myotonia, and pain. The effects of peptide toxins, especially those isolated from spider venom, have shed light on the structure–function relationship of these channels. However, most of these toxins have not been analyzed in detail. In particular, the bioactive faces of these toxins have not been determined. Jingzhaotoxin (JZTX)-V (also known as β-theraphotoxin-Cj2a) is a 29-amino acid peptide toxin isolated from the venom of the spider C*hilobrachys jingzhao*. JZTX-V adopts an inhibitory cysteine knot (ICK) motif and has an inhibitory effect on voltage-gated sodium and potassium channels. Previous experiments have shown that JZTX-V has an inhibitory effect on TTX-S and TTX-R sodium currents on rat DRG cells with *IC*_50_ values of 27.6 and 30.2 nM, respectively, and is able to shift the activation and inactivation curves to the depolarizing and the hyperpolarizing direction, respectively. Here, we show that JZTX-V has a much stronger inhibitory effect on Na_V_1.4, the isoform of voltage-gated sodium channels predominantly expressed in skeletal muscle cells, with an *IC*_50_ value of 5.12 nM, compared with *IC*_50_ values of 61.7–2700 nM for other heterologously expressed Na_V_1 subtypes. Furthermore, we investigated the bioactive surface of JZTX-V by alanine-scanning the effect of toxin on Na_V_1.4 and demonstrate that the bioactive face of JZTX-V is composed of three hydrophobic (W5, M6, and W7) and two cationic (R20 and K22) residues. Our results establish that, consistent with previous assumptions, JZTX-V is a Janus-faced toxin which may be a useful tool for the further investigation of the structure and function of sodium channels.

## 1. Introduction

Voltage-gated sodium channels (VGSCs), the current of which was discovered by Nobel laureates Hodgkin and Huxley in 1952, are critical elements of cellular function because they participate in the generation and propagation of action potentials in excitable cells, such as muscle cells and neuron [1,2,3,4,5]. The crystal structure of the homotetrameric bacterial sodium (Na) channel Na_V_Ab was solved by Payandeh *et al.* at high resolution (2.7 Å) in 2011 and provided insights into the atomic structure of mammalian sodium channels at some level [6]. VGSCs are composed of pore-forming α subunits associated with up to four known β subunit variants [7,8]. The α subunits are classified according to sequence homology as Na_V_1.1 to Na_V_1.9 (a less-defined subunit is called NaX [9,10]) and are further divided by their sensitivity to tetrodotoxin (TTX). Na_V_1.8, and Na_V_1.9 are TTX-resistant (TTX-R), and the remaining α subunits are TTX-sensitive (TTX-S) [10]. Subtype localization varies: Na_V_1.1, Na_V_1.2, and Na_V_1.3 are principally found in the central nervous system, whereas Na_V_1.6, Na_V_1.7, Na_V_1.8, and Na_V_1.9 are mostly distributed in the peripheral nervous system [10,11,12,13,14,15,16,17,18,19]. Na_V_1.4 can be found in skeletal muscle, and Na_V_1.5 is present predominantly in cardiac muscle [20,21].

The proper activity of these channels is crucial to the initiation of action potentials, which ultimately leads to muscle contraction or neuronal firing. Mutations in the SCN4A gene (sodium channel, voltage-gated, type IV, alpha subunit) encoding the human skeletal muscle Na_V_1.4 channel cause five different skeletal muscle disorders: potassium-aggravated myotonia (PAM), paramyotonia congenita (PMC), hyperkalemic periodic paralysis (hyperPP), hypokalemic periodic paralysis (HypoPP), and a form of congenital myasthenic syndrome (CMS) [22].

Spiders are known for their ability to produce complex venoms for predation and defense, which contributes to them being the most numerous order of terrestrial predators [23,24]. Decades of research has revealed that spider venoms are complex chemical mixtures, and a group of venom components are small disulfide-rich peptides [25]. According to published data, over 42,700 species of spiders have been defined, and a single form of venom can contain as many as 1000 peptides, most of which have not been well-characterized or analyzed [25,26,27]. ArachnoServer, a manually managed database, provides detailed information about well-defined spider venom peptides [28]. As crucial components for the development of action potentials, VGSCs are one of the common targets of animal venoms and plant neurotoxins. Toxins from various organisms have been used to describe eight different receptor sites on the α subunits of VGSCs, all of which are linked to specific effects on channel function [27]. For most of these toxins, the precise pattern of subtype selectivity is either unknown or, at best, fragmentary.

NaSpTx families 1–12, related spider venom peptides that act on VGSCs, have been well-defined recently in terms of their activities and sequence similarities. Among these 12 families, NaSpTx family 3 has 14 members that are 29–32 peptides long, with highly conserved sequences in the *C*-terminal region [29]. Jingzhaotoxin (JZTX)-V (also known as β-theraphotoxin-Cj2a), is a 29-amino acid peptide derived from the venom of *Chilobrachys jingzhao*, from which more than 18 peptides were discovered and defined. MALDI-TOF (Matrix-Assisted Laser Desorption/Ionization Time of Flight) mass spectrometry and complementary (c) DNA sequence analysis have detected amidation at the *C*-terminal residue of JZTX-V, and partial reduction and sequence analysis have identified three disulfide bonds. The linkage pattern of the disulfide bonds is I–IV, II–V, and III–IV, which defines JZTX-V as a member of classic ICK (Inhibitor Cysteine Knot) family. JZTX-V inhibits neuronal TTX-S and TTX-R Na channels in rat dorsal root ganglion (DRG) neurons with *IC*_50_ values of 27.6 and 30.2 nM, respectively [30]. However, little is known about the mechanism of inhibitory action of JZTX-V, especially regarding the specific sites involved in this interaction. In this manuscript, we investigated the toxin’s selectivity for four human (h) or rat (r) VGSC subtypes (hNa_V_1.3, rNa_V_1.4, hNa_V_1.5, and hNa_V_1.7) and the specific sites involved in the action of JZTX-V on Na_V_1.4. Our data indicate that JZTX-V has a much stronger effect on Na_V_1.4 over other tested subtypes, and the bioactive face of this toxin is composed of three hydrophobic (W5, M6, and W7) and two cationic (R20, K22) residues.

## 2. Results and Discussion

### 2.1. Selectivity of JZTX-V for Voltage-Gated Sodium Channel Subtypes

**Figure 1:**
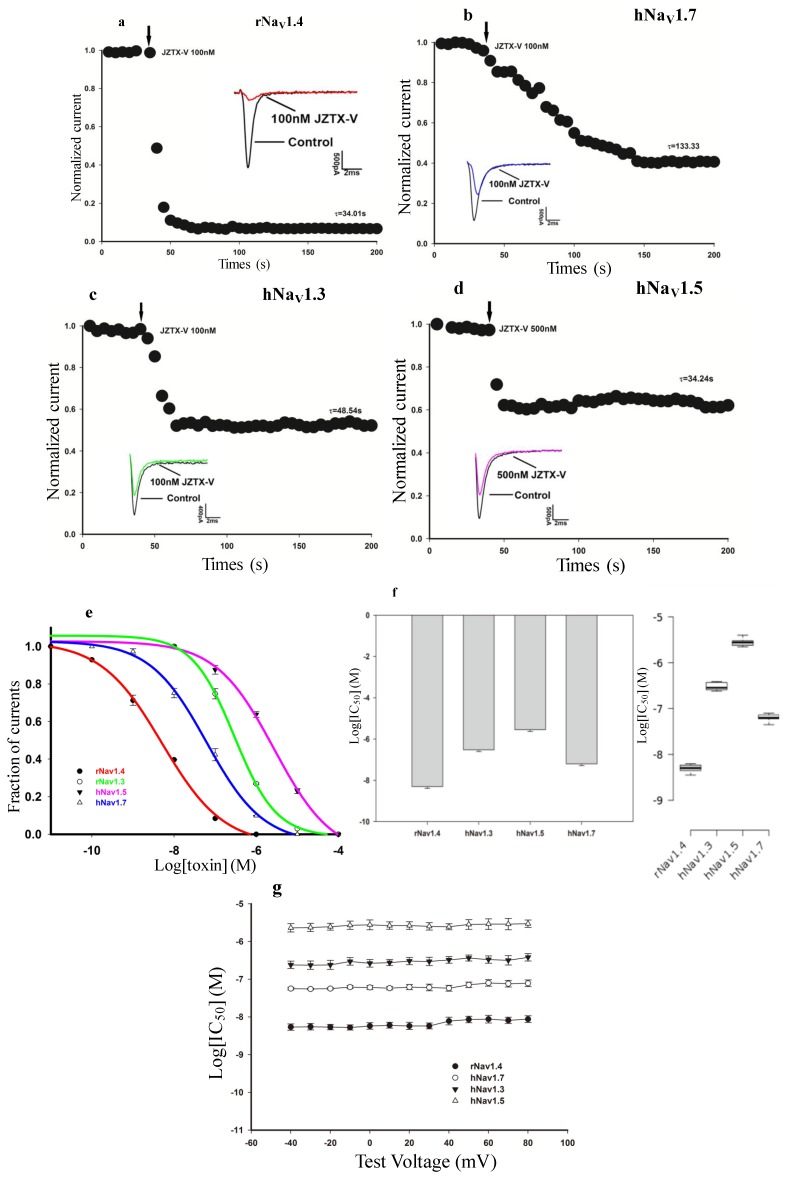
JZTX-V (Jingzhaotoxin-V) differentially blocks four wild-type voltage-gated sodium channel isoforms expressed in HEK (human embryonic kidney) 293 cells. Rat rNa_V_1.4 (rat voltage-gated sodium channels subtype 1.4) (**a**); human hNa_V_1.7 (human voltage-gated sodium channels subtype 1.7) (**b**); hNa_V_1.3 (**c**) and hNa_V_1.5 (**d**) sodium channels were exposed to JZTX-V. All inward current traces (inset) were elicited by a 50-ms depolarizing potential of −10 mV from a holding potential of −100 mV every 5 s. Data are normalized to the maximum peak current amplitude. After treatment with 100 nM or 500 nM JZTX-V, inward current of rNa_V_1.4 (**a**) was inhibited nearly completely, and approximately 45%, 35%, and 30% of hNa_V_1.7 (**b**); hNa_V_1.3 (**c**) and hNa_V_1.5 (**d**) inward currents were inhibited, respectively; The time constants of inhibition (inset) were 34.01 ± 6.81 s (rNa_V_1.4, **a**), 133.33 ± 27.96 s (hNa_V_1.7, **b**), 48.54 ± 11.35 s (hNa_V_1.3, **c**) and 34.24 ± 8.13 s (hNa_V_1.5, **d**), respectively; (**e**) Dose-response inhibitory curves for wild-type rNa_V_1.4, hNa_V_1.3, hNa_V_1.5, and hNa_V_1.7 exposed to JZTX-V. Each data point is shown as the mean ± standard error (S.E.) from three to six experimental cells. Data points were fitted with the Hill equation as described in the Experimental Section, yielding the following *IC*_50_ values: hNa_V_1.3, 292 *±* 61 nM; rNa_V_1.4, 5.12 *±* 0.87 nM; hNa_V_1.5, 2.7 *±* 0.779 μM; and hNa_V_1.7, 61.7 *±* 1.2 nM; (**f**) The column graph (left) and the box plot (right) show the *IC*_50_ values of JZTX-V on different VGSC subtypes as described; (**g**) *IC*_50_ values of JZTX-V on different VGSC subtypes were determined at a range of different depolarizing potentials (from −40 mV to +80 mV). Data are expressed as mean ± S.E.

Previous work discovered that JZTX-V inhibited TTX-S and TTX-R sodium currents in rat DRG neurons with *IC*_50_ values of 30.2 nM and 27.6 nM, respectively [30]; however, the effect of JZTX-V on other VGSC subtypes remains unknown. Therefore, we chose to investigate the ability of JZTX-V to block currents generated by four human (h) or rat (r) VGSC subtypes (hNa_V_1.3, rNa_V_1.4, hNa_V_1.5, and hNa_V_1.7) expressed in human embryonic kidney (HEK) 293 cells. Using whole-cell voltage clamp recording techniques, currents were elicited by a 50-ms depolarizing potential of −10 mV from a holding potential of −100 mV every 5 s. As shown in Figure 1, toxin treatments variably inhibit the peak current of four VGSC subtypes expressed in HEK 293 cells in a time-dependent manner. We observed that 100 nM JZTX-V could rapidly and completely inhibit wild-type rNa_V_1.4 (*n* = 6), whereas treatment with 500 nM JZTX-V decreased activation of wild-type hNa_V_1.5 by only 43% ± 6.5% (*n* = 8). The time constants for these inhibitions were 34.01 ± 6.81 and 34.24 ± 8.13 s, respectively (Figure 1a,d). However, the inhibition was much slower for wild type hNa_V_1.7 after treatment with 100 nM JZTX-V (τ = 133.33 ± 27.96 s, *n* = 6, Figure 1b). Moreover, the time constant for the inhibition of hNa_V_1.3 was 48.54 ± 11.35 s (*n* = 6, Figure 1c). Dose-response curves for Na_V_1 subtypes tested are summarized in Figure 1e and consist of at least three data points for each concentration. Fitting the data with the Hill equation yielded the following *IC*_50_ values: hNa_V_1.3, 292 ± 61 nM; rNa_V_1.4, 5.12 ± 0.87 nM; hNa_V_1.5, 2.7 ± 0.779 μM; and hNa_V_1.7, 61.7 ± 1.2 nM. In order to investigate whether the inhibition of JZTX-V is voltage-dependent, we tested and calculated the *IC*_50_ values of JZTX-V against the above four VGSC subtypes using the same method with a range of different depolarizing potentials (from −40 mV to +80 mV), which are depicted in Figure 1g. The results show that the inhibitions of JZTX-V on rNav1.4, hNav1.5, hNav1.3 and hNav1.7 lack voltage-dependence. These data indicate that Na_V_1.4, a skeletal muscle subtype, is the most sensitive to JZTX-V among the four tested VGSC isoforms and under different depolarizing potentials. This surprising finding has updated the discovery that JZTX-V preferentially inhibits the TTX-S and TTX-R VGSCs expressed in rat DRG cells. Moreover, the data show that JZTX-V is at least 500-fold more selective for inhibition of rNa_V_1.4 over the cardiac VGSC isoform Na_V_1.5 tested here. Compared with previously discovered toxins binding to Na_V_1.4 from spider venom, such as Phrixotoxin-3 (PaurTx3) from Phrixotrichus auratus, JZTX-V shows stronger inhibitory activity and more profound selectivity between Na_V_1.4 and Na_V_1.5 [31].

### 2.2. Effects of Subsaturating Concentrations of JZTX-V on Activation and Inactivation Properties of the Wild-Type VGSC Subtype Na_V_1.4

Furthermore, we wondered whether JZTX-V was acting as a simple pore blocker or as a gating modifier at subsaturating concentrations which did not inhibit the sodium current completely. Modification of the voltage-dependence of channel activation and inactivation can be a crucial feature of peptide toxins on VGSCs. Many toxins from spiders, such as Hm-1, Hm-2 and PaurTx3, are able to shift the steady-state activation or inactivation curves of Na_V_1.4 positively or negatively [31,32]. PaurTx3 has been found to be capable of inhibiting Na_V_1.4 with an *IC*_50_ of 288 ± 58 nM and shifting the I–V curve to more positive potentials, while it has no obvious effect on the inactivation rate [31]. Previous study has discovered that JZTX-V was able to shift the activation curves of TTX-S and TTX-R sodium currents on adult rat DRG neurons to depolarizing direction and the inactivation curves to the hyperpolarizing direction.

**Figure 2:**
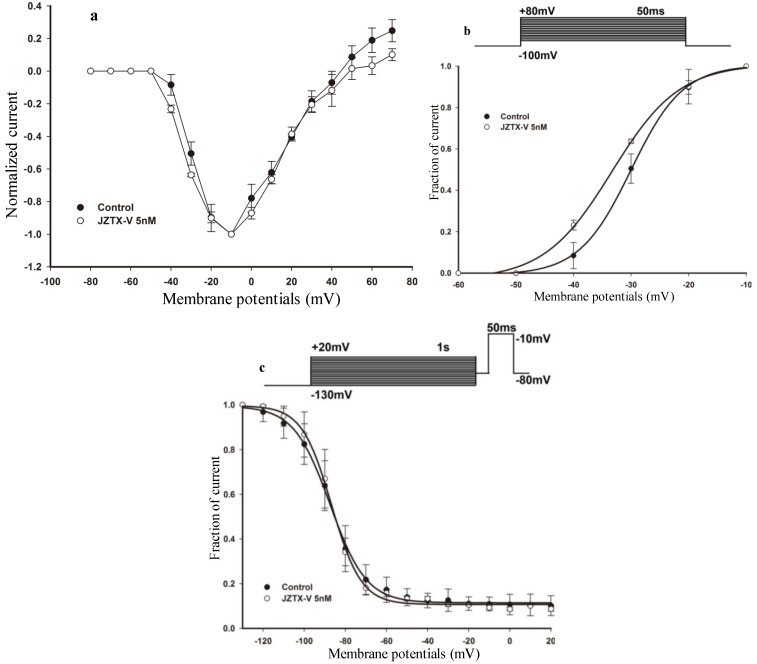
Effect of JZTX-V on rNa_V_1.4 channels. (**a**) Current-voltage (I–V) relationship of sodium currents before (solid circle) and after (open circle) treatment with 5 nM JZTX-V at holding potential for approximately 10 min. All the currents were normalized to the maximum amplitude of peak current; (**b**) Voltage-dependence of steady-state activation was estimated using a standard protocol. The above diagram shows the protocol for steady-state activation analysis. The currents data used for (**a**) and (**b**) were induced by 50-ms depolarizing steps to various potentials from a holding potential of −100 mV. Test potentials ranged from −80 mV to +80 mV in 10-mV increment. HEK 293 cells expressing rNa_V_1.4 channels were incubated in 5 nM JZTX-V for 10 min at a holding potential of −100 mV to allow toxin binding. JZTX-V (5 nM) shifted the steady-state activation hyperpolarizingly; (**c**) Voltage-dependence of steady-state inactivation was estimated using a standard double-pulse protocol. The above diagram shows the protocol for steady-state inactivation analysis. The rNa_V_1.4 currents were induced by a 50-ms depolarizing potential of −10 mV from various prepulse potentials for 1 s which ranged from −130 to +20 mV with a 10 mV increment. JZTX-V (5 nM) had no obvious effect on steady-state activation. Data points (mean ± standard error) from activation and inactivation kinetics were fit with Boltzmann function.

Therefore, we investigated the effects of subsaturating toxin concentrations on the voltage-dependence of channel steady-state activation and inactivation. The current-voltage (I–V) relationships were tested for rNa_V_1.4 current using step depolarization ranging from −80 mV to 80 mV, in 10 mV increments from a holding potential of −100 mV. Figure 2a shows that, under control conditions, the threshold of initial channel activation ranged between −40 and −30 mV, and the largest peak current was elicited between −20 and 0 mV. In the presence of 5 nM JZTX-V, the current voltage (I–V) curve was shifted hyperpolarizingly. Viewed from the steady-state activation curves before (solid circle, Figure 2b) and after (open circle, Figure 2b) the treatment with 5 nM JZTX-V at holding potential for 10 min, the midpoint voltage of rNa_V_1.4 activation was shifted negatively by 3 mV from −30.02 ± 0.25 mV (control) to −33.36 ± 0.75 mV (after treatment with 5 nM JZTX-V at holding potential for approximately 10 min), which is in contrast to the character of PaurTx3. Meanwhile, the slope factor of voltage-dependence activation curves was changed from 4.51 ± 0.26 mV (control, solid circle, Figure 2b) to 5.88 ± 0.71 mV (after treatment with 5 nM JZTX-V at holding potential for approximately 10 min, open circle, Figure 2b).

We also asked whether subsaturating concentrations of JZTX-V altered the voltage-dependence of steady-state inactivation. Steady-state inactivation is another important property of VGSCs that can modulate the excitability of neurons, can be modified by scorpion and spider toxins, and contributes to the inhibitory action of therapeutic agents such as lidocaine. Therefore, we investigated the effect of JZTX-V on steady-state inactivation of Na_V_1.4 using a standard two-pulse protocol. The rNa_V_1.4 currents were induced by a 50-ms depolarizing potential of −10 mV from various prepulse potentials for 1 s which ranged from −130 to +20 mV with a 10 mV increment. As shown in Figure 2c, under control conditions, the calculated midpoint of steady-state inactivation was −87.41 ± 0.51 mV. After treatment with 5 nM JZTX-V for approximately 10 min, the estimated midpoint value was −86.71 ± 0.53 mV. The midpoint voltage of rNa_V_1.4 steady-state inactivation was shifted positively ~1 mV, however, the slope factors of steady-state inactivation curves were changed from –8.81 ± 0.44 mV (control, solid circle, Figure 2c) to −7.21 ± 0.46 mV (after treatment with 5 nM JZTX-V, open circle, Figure 2c) Thus, these data indicate that subsaturating concentrations of JZTX-V have not obvious effect on steady-state activation and inactivation of the residual currents conducted by skeletal muscle VGSCs, but it can change the slope factor of steady-state activation and inactivation curves.

### 2.3. Synthesis and Structural Integrity of JZTX-V

**Figure 3:**
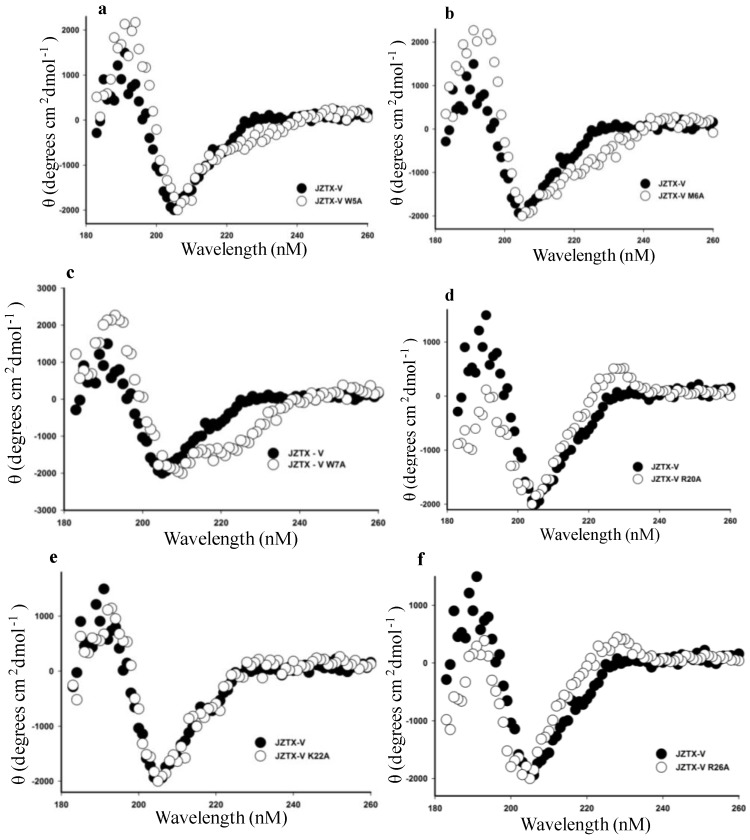
CD (circular dichroism) spectra of wild-type JZTX-V and its mutants. (**a**–**f**) Comparison of the CD spectra of wild-type JZTX-V and its mutants (**a**, W5A; **b**, M6A; **c**, W7A; **d**, R20A; **e**, K22A; and **f**, R26A). Toxins were tested from 260 nm–180 nm in 0.01 M sodium phosphate solution (pH 7.0) at room temperature. θ, mean residue ellipticity.

Previous studies have indicated that the hydrophobic patch and a group of positive residues of Protoxin-II (ProTx-II), which is a 30-amino acid peptide from the tarantula *Thrixopelma pruriens* that shares 72% sequence identity with JZTX-V, play a critical role in binding to Na_V_1.5 and are responsible for the pharmacological features of ProTx-II [30,33]. Due to the high homology with ProTx-II, we proposed that JZTX-V adopts the same bioactive face in binding to Na_V_1.4. In our study, we tried to investigate and disclose the amino acid residues of JZTX-V that play a crucial function in binding to rNa_V_1.4 by introducing site mutations into every amino acid position except for cysteine, but we were able to mutate only 19 residues successfully. First, the structure integrity of 19 mutant toxins was detected by measuring the CD spectrum from 260–180 nM in 0.01 M sodium phosphate solution (pH 7.0) at room temperature. A circular dichroism spectrum overlapping that of the wild-type toxin is characteristic of a normal fold. Figure 3 demonstrates that the CD spectra of JZTX-V mutants overlapped nearly completely with that of the synthesized wild-type JZTX-V, indicating that the mutation of the residues had no effect on the peptide’s molecular structure. Second, we determined that the high-performance liquid chromatography (HPLC) retention time of mutants is similar to wild-type. Third, a molecular weight was analyzed by MALDI-TOF mass spectrometry which should be identical to the calculated mass of oxidized toxin. With the exception of the HPLC profile of R26A of JZTX-V, all of the profiles exhibited HPLC retention times that were similar to the wild-type JZTX-V (data not shown). The R26A of JZTX-V HPLC profile showing a heterogeneous population of peptide forms, which indicates a folding aberration, means R26A mutant is not suitable for further function research. 

**Figure 4:**
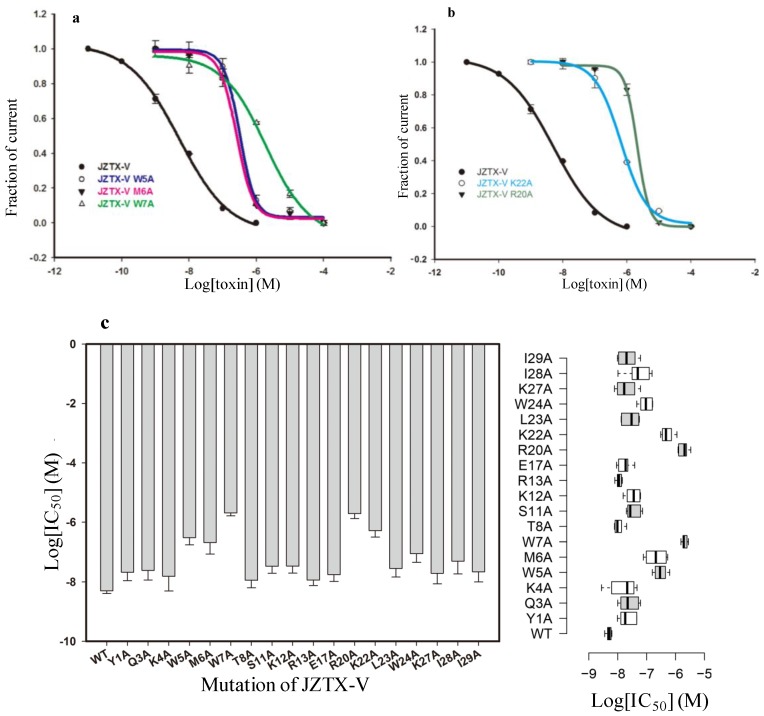
Inhibitory effects of wild-type and mutant JZTX-V on rNa_V_1.4 current. (**a**) Dose-response inhibitory curves for wild-type and mutant JZTX-V (W5A, M6A, and W7A) on wild-type rNa_V_1.4. Compared with wild-type JZTX-V, W5A, M6A, and W7A mutants significantly reduce binding affinity to the rNa_V_1.4 channel; (**b**) Dose-response inhibitory curves for wild-type and mutant JZTX-V (K22A and R20A) on wild-type rNa_V_1.4. Compared with wild-type JZTX-V, K22A and R20A mutants significantly reduce affinity to rNa_V_1.4; (**c**) The column graph (**left**) and the box plot (**right**) show the *IC*_50_ values of JZTX-V and its mutants, as described.

### 2.4. Functional Characterization of Mutant Forms of JZTX-V

After purification, we characterized the interaction of each mutant toxin with Na_V_1.4. Under whole-cell patch clamp configuration, rNa_V_1.4 current was elicited by a 50-ms depolarizing potential of −10 mV from a holding potential of −100 mV every 5 s. According to a structural model of JZTX-V based on the structure of κ-theraphotoxin-Gr3a (VSTx1), a large hydrophobic patch composed of three aromatic residues (W5, W7, and W24) and two aliphatic residues (M6 and L23) were observed on the surface of the structural model [34,35]. In Figure 4a, compared to recombinant JZTX-V, alanine mutations of 3 hydrophobic residues (W5A, M6A, and W7A) were able to alter toxin affinity toward Na_V_1.4 significantly. Fitting a Hill equation to the data in Figure 4a yielded *IC*_50_ values of 0.313 ± 0.26 (W5A), 0.252 ± 0.45 (M6A), and 1.92 ± 0.31 μM (W7A) (Figure 4a,c), suggesting that the three mutants reduce JZTX-V binding affinity by 61-, 49- and 375-fold, respectively. In particular, the W7A mutation nearly abolished the binding affinity of JZTX-V on rNa_V_1.4. However, alanine mutations of the other two hydrophobic residues (W24 and L23) failed to reduce toxin-binding affinity. Along with the structural model of JZTX-V, these results revealed that the central portion of the hydrophobic patch (W5A, M6A, and W7A) plays a more vital role in the interaction process, compared with the two hydrophobic residues (W24 and L23) lying outside.

We further investigated whether positively charged residues are involved in JZTX-V action, which has also been discovered on ProTx-II. In Figure 4b, compared to recombinant JZTX-V, alanine mutations of two positively charged residues (R20A and K22A) were able to alter toxin affinity toward Na_V_1.4 significantly and yielded *IC*_50_ values of 2.1 ± 0.28 (R20A) and 0.659 ± 0.011 (K22A) μM, respectively (Figure 4b,c). These results show that the mutations of these two positively charged residues were able to reduce JZTX-V binding affinity by 410- and 128-fold, respectively. The changed binding affinities of the other two residues (R20A and K22A) were due to the loss of positive charge. Viewed from the molecular model of JZTX-V, R20 and K22 residues are located to the left of the hydrophobic patch. More interestingly, previous research has predicted that the positively charged residues on the surface of JZTX-V may interact with the negatively charged phospholipids on the basis of phospholipid-binding experiments. The discovery of decreased binding affinity caused by mutant R20A and K22A favors the previous presumption and proves that the positively charged residues are involved in the binding process to rNa_V_1.4. Data are expressed as mean ± S.E.

### 2.5. Discussion

#### 2.5.1. JZTX-V Is a Selective Antagonist of Voltage-Gated Sodium Channel Subtype Na_V_1.4

JZTX-V, a 3.6 kDa neurotoxin isolated from the venom of *Chilobrachys jingzhao*, has been proven to be capable of inhibiting TTX-S and TTX-R sodium currents in rat DRG neurons with *IC*_50_ values of 27.6 and 30.2 nM, respectively [30]. Our current work showed that JZTX-V is one of the most potent inhibitors of Na_V_1.4, with an *IC*_50_ of 5.21 nM, which is more than 30-fold lower than the reported *IC*_50_ values of β-theraphotoxin-Ps1a (PaurTx3, 288 ± 58 nM) and β-theraphotoxin-Gr1b (GsAF-I, 330 ± 30 nM), suggesting that JZTX-V is a strong selective antagonist of Na_V_1.4.

#### 2.5.2. Effect of JZTX-V on Voltage-Gated Sodium Channel Subtype Na_V_1.4

Previous study revealed that JZTX-V shifts the half-maximal activation potential of TTX-S and TTX-R sodium currents in the depolarizing direction and the half-maximal steady-state inactivation potential in the hyperpolarizing direction. In contrast to its effect on TTX-S and TTX-R sodium currents of adult rat DRG neuron, JZTX-V is able to shift the steady-state activation curve hyperpolarizingly (~3 mV). However, this shift in the voltage-dependence of activation after treatment with JZTX-V is marked by a change in the slope factor of the activation curve. The mechanism for this change in the slope factor of activation curve needs further investigation. The possible explanation for this change might be that the cooperativity between the four S4 segments might be changed or it might be associated with additional binding sites [36]. Moreover, the discrepancy with previous results may be partly due to the difference in expression backgrounds for these channels. The sodium channels associate with variant protein partners, such as β subunits, that play a role in regulating channel trafficking and gating, which means sodium channel properties is modulated in a cell-type specific manner [37,38,39]. Recently, β4 subunit have been proven to dramatically change the sensitivity of Na_V_1.2 subtype to spider toxin ProTx-II, which serves as a solid evidence that β4 subunit is involved in the interaction between the toxin and its pharmacological target [40]. It is reasonable to believe that the cell background, especially the expression level of VGSC associated protein partners, can alter the effect of toxins on its targeted VGSCs.

#### 2.5.3. The Bioactive Surface of JZTX-V on Voltage-Gated Sodium Channel Subtype Na_V_1.4

**Figure 5:**
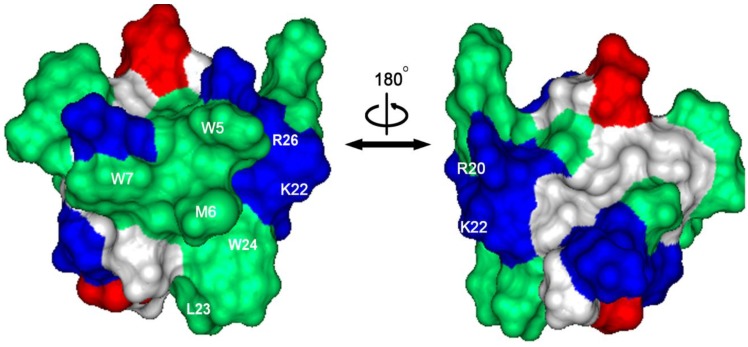
The surface profile of JZTX-V. Left and right structures are rotated 180° relative to one another about a vertical axis. Hydrophobic residues are green in color; basic residues are blue in color, and acidic resides are red in color. Five hydrophobic residues (W5, M6, W7, L23 and W24) and three charged residues (R20, K22, and R26) are located on the right side of the hydrophobic patch

Known from its structural model, which is based on the molecular structure of VSTx1, JZTX-V adopts a Janus-faced surface profile with two functional bioactive face (Figure 5), in which a large hydrophobic patch consisting of three aromatic residues and two aliphatic residues (W5, M6, W7, L23, and W24) is formed on the surface, and four positively charged residues (R20, K22, R26, and K27) surround the hydrophobic patch [41]. Previous phospholipid membrane-binding experiments indicated that the basic residues on the surface of JZTX-V play a critical role in the binding to VGSC [30]. To investigate which residue is critical for JZTX-V binding to Na_V_1.4, each non-cysteine residue was mutated to alanine, and 19 mutants of JZTX-V, all of which exhibited CD spectra identical to that of the wild-type toxin, were synthesized and folded successfully by solid-phase peptide synthesis. Based on our electrophysiological experiments with the wild-type and 19 mutants of JZTX-V, two consequential patches have been detected on the surface of JZTX-V.

The W5, M6, and W7 residues, which form a hydrophobic patch on the surface of JZTX-V, were shown to play an important role in JZTX-V binding to Na_V_1.4. Compared with wild-type, the *N*-terminal mutation W7A reduced binding affinity more than 300-fold. Similarly, the cluster of hydrophobic residues on the surface of ProTx-II have been shown to be involved in binding to Na_V_1.5 [30,33].

In addition to the hydrophobic amino acids of JZTX-V, two positively charged residues, R20 and K22, are critical for the binding of JZTX-V to Na_V_1.4. K22A and R20A mutations induce strong decreases in binding affinity between the toxin and Na_V_1.4 (128- and 410-fold, respectively), which suggests that an electrostatic interaction with the channel or the phospholipid head groups may be involved in the binding process. This is consistent with previous results from the phospholipid membrane binding experiment, which suggest an electrostatic interaction between the basic residues of JZTX-V and the negatively charged phospholipids. To further investigate whether JZTX-V interacts with the lipid bilayer before accessing a target wholly or partially within the membrane, a group of experiments involving mutations of Na_V_1.4 is in process.

#### 2.5.4. Comparison with Other Spider Toxins Which Have Effects on Voltage-Gated Sodium Channel Subtype Na_V_1.4

After searching the spider venom database ArachnoServer, six toxins from different kinds of spider venom have been found to have an inhibitory effect on Na_V_1.4, all of which have been identified and classified as ICK toxins. Firstly, two newly found toxins isolated from the venom of the crab spider *Heriaeus melloteei*, Hm-1 and Hm-2, consisting of 37 and 40 amino acid residues, respectively, have been found to inhibit Na_V_1.4 (expressed with β1 subunit in oocytes) with *IC*_50_ values of 336.4 nM and 154.8 nM, respectively. These two toxins did not shift the voltage-dependence of activation and the kinetics of fast inactivation of the Na_V_1.4 current [42]. However, these toxins can shift the steady-state inactivation negatively. Secondly, ceratotoxin-1 and ceratotoxin-2, isolated from the venom of *Ceratogyrus marshalli* and differing by only one amino acid, were proven to have weak inhibitory effects on Na_V_1.4 (expressed with β1 subunit in oocytes), with *IC*_50_ values of 880 nM and 400 nM, respectively. Interestingly, ceratotoxin-1 and ceratotoxin-2 were found to have a stronger effect on Na_V_1.2 with *IC*_50_ values of 3 nM and 8 nM, respectively. Thirdly, PharTx-3, a toxin isolated from the venom of *Paraphysa scrofa* with 34 amino acid residues, was one of the most potent peptide modulators of Na_V_1.2, with an *IC*_50_ value of 0.6 nM. PharTx-3 was able to shift the voltage dependence of voltage-gated sodium channel subtype Na_V_1.2 activation to more depolarized potentials. However, it has a weak effect on Na_V_1.4, with an *IC*_50_ value of 288 nM. Compared with previously discovered toxins that have an inhibitory effect on Na_V_1.4, JZTX-V (1) is one of the most potent peptide inhibitors of Na_V_1.4, with an *IC*_50_ value of 5.21 nM; and (2) can induce a small hyperpolarizing shift marked by a prominent change in the slope of the curve and has no obvious effect on the inactivation curve of Na_V_1.4.

## 3. Experimental Section

### 3.1. Peptide Synthesis and Oxidation

The wild-type and mutants of JZTX-V were synthesized through solid peptide synthesis as described by Zeng *et al.* [43].

### 3.2. Transient Transfection

All the voltage gated sodium channels subtypes (hNa_V_1.3, rNa_V_1.4, hNa_V_1.5 and hNa_V_1.7), along with β1 subunit to increase the current density and green fluorescent protein to serve as the report protein, were individually transiently transfected into HEK 293 cells using the lipofectamine 2000 (Invitrogen, Carlsbad, CA, USA) according to its manufacturer’s instructions. HEK293 cells were cultured under standard cell culture condition (5% CO_2_; 37 °C) in DMEM supplemented with 10% FBS. At 36–72 h after transfection, cells with green fluorescence were selected for whole-cell patch-clamp recording at room temperature. The pipette solution contained (in mM): 140 CsF, 10 NaCl, 10 HEPES and 1 EGFA, pH7.3. The bathing solution contained (in mM): 140 NaCl, 10 HEPES, 3 KCl, 1 MgCl_2_ and 1 CaCl_2_, pH7.3.

### 3.3. Whole-Cell Patch-Clamp Recording

Sodium currents were recorded on experimental cells using whole-cell patch-clamp techniques at room temperature (20–25 °C). Patch pipettes with DC resistance of 2~3 MΩ were fabricated from borosilicate glass tubing (VWR micropipettes; VWR Co., West Chester, PA, USA) using a two-stage vertical microelectrode puller (PC-10; Narishige, Tokyo, Japan) and fire-polished by a heater (Narishige, Tokyo, Japan). Whole-cell patch-clamp recordings were performed by an Axon 700B patch-clamp amplifier (Axon Instruments, Irvine, CA, USA). The P/4 protocol was used to subtract linear capacitive and leakage currents. Experimental data were acquired and analyzed using the programs Clampfit 10.0 (Axon Instruments, Irvine, CA, USA) and Sigmaplot 90 (Sigma, St. Louis, MO, USA).

### 3.4. Toxin Solutions and Bath Application

The stock solution of JZTX-V at 1mM was dissolved in double distilled water, and the solutions were stocked at −20 °C. Before use, the solution was diluted to the concentration of interest with fresh bathing solution. Toxin was introduced into the recording chamber (volume of 300 μL) and mixed by repeatedly pipetting 30 μL to achieve the demanded final concentration. The mixing process typically took ~5 s.

### 3.5. Data Analysis

Data were analyzed using the Clampfit (Molecular Devices, Irvine, CA, USA) and Sigmaplot 10.0 (Sigma, St. Louis, MO, USA) software programs. All data points are shown as mean ± S.E. n is the number of the separated experimental cells. Dose-response curves were fitted by using the following Hill logistic equation: *y* = 1 − (1 − *f*_max_)/(1 + ([*T*x]/*IC*_50_)*^n^*) where n is an empirical Hill coefficient and *f*_max_ is the fraction of current resistant to inhibition at high except where indicated otherwise. The Hill coefficient was set to 1 except where indicated. The box plots were generated at http://boxplot.tyerslab.com [44]. In the box plots, center lines show the medians; box limits indicate the 25th and 75th percentiles as determined by R software; whiskers extend 1.5 times the interquartile range from the 25th and 75th percentiles, outliers are represented by dots. Time courses were fitted by using single exponential decay function.

## 4. Conclusions

In this study, we provide solid evidence that (1) JZTX-V is able to inhibit rat Na_V_1.4 current with an *IC*_50_ value of 5.12 nM, which is 500-fold more selective over the cardiac VGSC isoform Na_V_1.5; (2) the bioactive surface of JZTX-V contains important positively charged residues (R20 and K22) and hydrophobic residues (W5, M6, and W7); and (3) homologous toxins such as JZTX-V and ProTx-II may present the same interaction surface to their respective targeted VGSC isoforms.
